# Racemic Salsolinol and its Enantiomers Act as Agonists of the μ-Opioid Receptor by Activating the Gi Protein-Adenylate Cyclase Pathway

**DOI:** 10.3389/fnbeh.2016.00253

**Published:** 2017-01-23

**Authors:** Pablo Berríos-Cárcamo, María E. Quintanilla, Mario Herrera-Marschitz, Vasilis Vasiliou, Gerald Zapata-Torres, Mario Rivera-Meza

**Affiliations:** ^1^Program of Molecular and Clinical Pharmacology, Faculty of Medicine, Institute of Biomedical Sciences, University of ChileSantiago, Chile; ^2^Department of Environmental Health Sciences, Yale School of Public HealthNew Haven, CT, USA; ^3^Department of Analytical and Inorganic Chemistry, Faculty of Chemical and Pharmaceutical Sciences, University of ChileSantiago, Chile; ^4^Department of Pharmacological and Toxicological Chemistry, Faculty of Chemical and Pharmaceutical Sciences, University of ChileSantiago, Chile

**Keywords:** salsolinol, μ-opioid receptor, naltrexone, β-arrestin, molecular docking

## Abstract

**Background**: Several studies have shown that the ethanol-derived metabolite salsolinol (SAL) can activate the mesolimbic system, suggesting that SAL is the active molecule mediating the rewarding effects of ethanol. *In vitro* and *in vivo* studies suggest that SAL exerts its action on neuron excitability through a mechanism involving opioid neurotransmission. However, there is no direct pharmacologic evidence showing that SAL activates opioid receptors.

**Methods**: The ability of racemic (R/S)-SAL, and its stereoisomers (R)-SAL and (S)-SAL, to activate the μ-opioid receptor was tested in cell-based (light-emitting) receptor assays. To further characterizing the interaction of SAL stereoisomers with the μ-opioid receptor, a molecular docking study was performed using the crystal structure of the μ-opioid receptor.

**Results**: This study shows that SAL activates the μ-opioid receptor by the classical G protein-adenylate cyclase pathway with an half-maximal effective concentration (EC_50_) of 2 × 10^−5^ M. The agonist action of SAL was fully blocked by the μ-opioid antagonist naltrexone. The EC_50_ for the purified stereoisomers (R)-SAL and (S)-SAL were 6 × 10^−4^ M and 9 × 10^−6^ M respectively. It was found that the action of racemic SAL on the μ-opioid receptor did not promote the recruitment of β-arrestin. Molecular docking studies showed that the interaction of (R)- and (S)-SAL with the μ-opioid receptor is similar to that predicted for the agonist morphine.

**Conclusions**: It is shown that (R)-SAL and (S)-SAL are agonists of the μ-opioid receptor. (S)-SAL is a more potent agonist than the (R)-SAL stereoisomer. *In silico* analysis predicts a morphine-like interaction between (R)- and (S)-SAL with the μ-opioid receptor. These results suggest that an opioid action of SAL or its enantiomers is involved in the rewarding effects of ethanol.

## Introduction

The mechanisms underlying the addictive properties of ethanol are still not fully understood since specific molecular targets explaining its pharmacological actions have not yet been identified. Unlike other drugs of abuse such as morphine, cocaine or nicotine that elicit their effects at micromolar blood concentrations (10^−6^ M; Jeffcoat et al., 1989; Glare and Walsh, 1991; Benowitz and Jacob, 1993), the pharmacological effects of ethanol are evident only at millimolar blood levels (10^−3^ M; Holford, 1987). The low potency of ethanol could be attributed to its molecular simplicity, hampering its binding with high affinity to any type of receptor. Nevertheless, a number of studies have shown that acetaldehyde, the primary metabolite of ethanol in the brain, is self-administered intracranially by rats at micromolar concentrations showing strong motivational and reinforcing effects (Rodd et al., 2005). Indeed, ethanol metabolism to acetaldehyde in the brain is required to exert ethanol’s reinforcing actions (Karahanian et al., 2011, 2015; Quintanilla et al., 2012; Israel et al., 2015; Peana et al., 2016).

In the brain, ethanol-derived acetaldehyde can condense non-enzymatically with dopamine to generate racemic (R/S)-salsolinol (SAL, 1-methyl-1,2,3,4-tetrahydro-6,7-dihydroxy-isoquinoline; Melchior and Collins, 1982). Studies by Rommelspacher et al. (1995) showed that levels of SAL in the blood of alcoholics were higher than those found in non-alcoholic individuals. The exposure of healthy individuals to an acute dose of ethanol results in an increase of SAL concentrations in blood and urine (Haber et al., 1996). Animal studies have shown that chronic ethanol administration to rats results in a significant increase of SAL levels in dopamine-rich areas of the brain such as striatum, limbic forebrain and hypothalamus (Sjöquist et al., 1982; Matsubara et al., 1987), yielding increased amounts of SAL (Rojkovicova et al., 2008). Behavioral studies performed in rats have shown that SAL administration results in major increases in voluntary ethanol intake (Myers and Melchior, 1977; Quintanilla et al., 2014, 2016), locomotor activity (Hipólito et al., 2010; Quintanilla et al., 2014) and conditioned place preference (Matsuzawa et al., 2000; Quintanilla et al., 2014). Microdialysis studies have shown that microinjections of SAL into the ventral tegmental area (VTA) of rats result in an increased release of dopamine in the nucleus accumbens (Deehan et al., 2013), a common hallmark shared by different drugs of abuse, including ethanol (Di Chiara and Imperato, 1988). Furthermore, SAL is self-administered intracranially by rats at concentrations ranging from 0.03 μM to 0.3 μM (Rodd et al., 2008; Deehan et al., 2013). These concentrations are 10–100 times lower than that required for acetaldehyde self-administration, suggesting that SAL is an active molecule involved in the rewarding effects of ethanol. Indeed, recent electrophysiology studies by Melis et al. (2015) showed that inhibition of dopamine synthesis by a tyrosine hydroxylase inhibitor fully abolishes the ability of ethanol (100 mM) and acetaldehyde (10 nM) to stimulate VTA dopaminergic neurons.

Experimental evidence suggests that the activating/rewarding properties of SAL are mediated by mechanisms involving μ-opioid receptors, although the levels at which this effect occurs is not known. Studies by Matsuzawa et al. (2000) showed that place preference conditioning elicited by SAL is significantly attenuated by the concomitant administration of the selective μ-opioid receptor antagonist β-funaltrexamine. Similar results were obtained by Hipólito et al. (2010), who found that the administration of β-funaltrexamine reduces the increase in locomotor activity elicited by the intra-VTA administration of SAL to rats. Recent studies by Quintanilla et al. (2014) showed that repeated systemic administration of racemic SAL (10 mg/kg, i.p.) led to a marked increase in voluntary ethanol intake in rats. This sensitization to ethanol intake was fully blocked by the concomitant intra-VTA administration of the opioid antagonist naltrexone, suggesting that the VTA is the primary site of action of SAL and its mechanism of action mediated by opioid receptors. It has been proposed that SAL could bind to μ-opioid receptors on GABAergic neurons of the VTA, inducing hyperpolarization, resulting in disinhibition and therefore activation of nearby dopaminergic neurons (Xie et al., 2013), resembling the action of opioid drugs (Johnson and North, 1992). In line with this view, electrophysiological studies performed in VTA-containing brain slices showed that SAL increased the firing of dopamine neurons by a μ-opioid/GABAergic combined mechanism, since the activating effects of SAL were blocked by both the μ-opioid antagonist naltrexone and the GABA_A_ receptor antagonist gabazine (Xie et al., 2012).

Despite early reports suggesting that SAL (10^−8^ to 10^−3^ M) can bind to opioid receptors (Fertel et al., 1980; Lucchi et al., 1982), there are no specific studies aimed at determining the intrinsic activity of SAL on μ-opioid receptors. We report here a light emission cell-based receptor assays describing the effect of SAL on μ-opioid receptors, based on the activation of the canonical signaling pathway associated to G protein. We also assessed the capacity of SAL to activate the G protein-independent signaling pathway associated with β-arrestin recruitment. We extended these *in vitro* studies to assess the action of the SAL stereoisomers (R)-SAL and (S)-SAL. To further understand the interaction of SAL with the μ-opioid receptor, we also investigated on molecular docking analyses, comparing (R)-SAL vs. (S)-SAL on their ability to bind to the active site of the mouse μ-opioid receptor, whose crystal structure was recently published (Huang et al., 2015).

## Materials and Methods

### Materials

Racemic SAL was purchased from Santa Cruz Biotechnology (Dallas, TX, USA), naltrexone was from Alfa Aesar (Ward Hill, MA, USA). Ammonium acetate was from Merck (Darmstadt, Germany) and triethylamine was from Sigma (St. Louis, MO, USA).

### Separation and Purification of (R) and (S)-Salsolinol

(R)-SAL and (S)-SAL were separated from the racemic solution by high-pressure liquid chromatography (HPLC) as described previously (Quintanilla et al., 2016). Briefly, a solution of (R/S)-SAL was injected into a NUCLEODEX β-cyclodextrin-modified column (Macherey-Nagel, Düren, Germany) kept at 20°C. The column was coupled to a LC-4C BAS amperometric detector (ED) set to a potential of 700 mV. The mobile phase, composed of volatile 100 mM ammonium acetate and 10 mM triethylamine (pH 4.0), was injected at a flow rate of 0.40 mL/min. Previous reports indicate that (S)-SAL enantiomer is the first to be eluted following similar chromatographic conditions (Deng et al., 1995; Naoi et al., 1996; Tóth et al., 2001; Quan et al., 2005; Rojkovicova et al., 2008; Lee et al., 2010). Once (R/S)-SAL was injected into the HPLC, the enantiomers were separated and collected according to their corresponding elution time (electrochemical detector disconnected). To check their purity, samples were reinjected onto the HPLC system. Each purified fraction was lyophilized at −54°C for 9 h for mobile phase elimination and dissolved in HCl 10^−5^ M, (pH 5.0). The concentration of purified samples was determined either by HPLC-ED or by absorbance at 290 nm, using a calibration curve with (R/S)-SAL as a standard. Samples were stored at −20°C in amber microtubes.

### Activation of μ-Opioid Receptors through the Gi Protein-Signaling Pathway

The intrinsic activity of the ligands was studied using commercially cell-based assays, composed by recombinant CHO-K1 cells that overexpress only the human μ-opioid receptor, detecting the levels of second messengers that reflect the activation of this receptor. To assess the activation of the μ-opioid receptor through the recruitment of G protein signaling pathway, we used the cyclic adenosine monophosphate (cAMP) Hunter^®^ eXpress G protein-coupled receptor (GPCR) Assay (DiscoverX, Freemont, CA, USA) following the manufacturer instructions. In this system, the endogenous cAMP competes with an exogenous cAMP coupled to a truncated β-galactosidase fragment (provided by the assay) for binding to a cAMP antibody. Only the unbound exogenous cAMP-β-galactosidase fragment binds to a complementary β-galactosidase fragment to form the active enzyme. The activity of β-galactosidase, measured by adding a chemiluminescent substrate, reflects proportionally the levels of cellular cAMP. (R)-SAL, (S)-SAL and (R/S)-SAL, dissolved in HCl 10^−5^ M, (pH 5.0) and including the adenylate cyclase activator forskolin (20 μM), were incubated at concentrations ranging from 1 × 10^−8^ M to 1 × 10^−3^ M with the CHO-K1 cells for 30 min. Morphine was incubated at concentrations ranging from 3 × 10^−9^ M to 1 × 10^−4^ M under the same conditions. Control cells were incubated only with forskolin. The resulting luminescence was measured with a microplate reader Synergy HT (Biotek, Winooski, VT, USA) or a microplate reader SpectraMax M3 (Molecular Devices, Sunnyvale, CA, USA). The experiment was repeated three times in duplicate for (R)-SAL, (S)-SAL and (R/S)-SAL and two times in duplicate for morphine.

In a subsequent experiment, the activation of the μ-opioid receptor by racemic SAL (150 μM) was studied after the addition of different concentrations (3 × 10^−10^ M to 10^−5^ M) of the antagonist naltrexone to the recombinant cells, dissolved in assay buffer, 30 min before to the addition of SAL. All measurements were expressed as relative luminescence to the controls with no ligand added. Each concentration of naltrexone was assayed once in duplicate. The antagonistic action of naltrexone was selective for μ-opioid receptor since the recombinant cells used in the study only overexpress this type of opioid receptor.

### Activation of μ-Opioid Receptors through the β-Arrestin Signaling Pathway

To assess the activation of the μ-opioid receptor through the recruitment of the β-arrestin signaling pathway, we used a PathHunter^®^ eXpress β-Arrestin GPCR chemiluminescent assay (DiscoverX) following the manufacturer instructions. In this system, the human μ-opioid receptor is fused to a small fragment of β-galactosidase (PK) and co-expressed in cells expressing a fusion protein of β-arrestin and the complementary fragment of β-galactosidase (EA). Activation of the μ-opioid receptor stimulates binding of β-arrestin to the PK-tagged receptor allowing the complementation of the two enzyme fragments of β-galactosidase. This action leads to an activation of the enzyme that can be measured using a chemiluminescent reagent resulting in luminescence, which is proportional to the recruitment of β-arrestin. DADLE ([D-Ala^2^, D-Leu^5^]-Enkephalin), (R/S)-SAL and morphine (dissolved in HCl 10^−5^ M, pH 5.0), were incubated at concentrations ranging from 1 × 10^−8^ to 1 × 10^−3^ M with the cells for 30 min. The experiment was repeated three times in duplicate for each concentration of ligand. The resulting luminescence was determined with a microplate reader Synergy HT (Biotek).

### Molecular Docking

Molecular docking simulations were performed using the crystal structure of the mouse μ-opioid receptor bound to the morphinan high-affinity agonist BU72 (Neilan et al., 2004), obtained from the protein data bank as a PDB file (ID: 5C1M; Huang et al., 2015). For the simulations, water molecules near the receptor were conserved, but BU72 and any other co-crystallized molecules were removed. After that, polar hydrogens were added to the protein coordinates (not resolved in the crystal structure). The studied molecules, morphine, (R)-SAL and (S)-SAL, were prepared and optimized to their energy minima using SPARTAN 10. Docking simulations were performed using Autodock Vina (Trott and Olson, 2010), which renders the ligand fully flexible, while to the target protein a rigid structure. The search space was 24 Å^3^ around a side-chain oxygen of Asp147, regarded as a critical residue for the binding of ligands to this receptor (Li et al., 1999; Manglik et al., 2012; Shim et al., 2013; Huang et al., 2015). The searching exhaustiveness was 800 (default 8). Ligand-protein interactions analyses and 3D figures were prepared using PyMOL (DeLano, 2002).

### Statistical Analyses

The half-maximal effective concentration (EC_50_) for each ligand and half-maximal inhibitory concentration (IC_50_) for naltrexone in the antagonist assay were determined by correlating the data to a non-linear equation of ligand concentration (log M) vs. response by three parameters. The fitting of the data to the correlation was assessed by its corresponding coefficient of determination (*R*^2^), using GraphPad Prism (San Diego, CA, USA).

## Results

### Activation of μ-Opioid Receptors by Racemic SAL through the Gi Protein-Signaling Pathway

**Figure 1:**
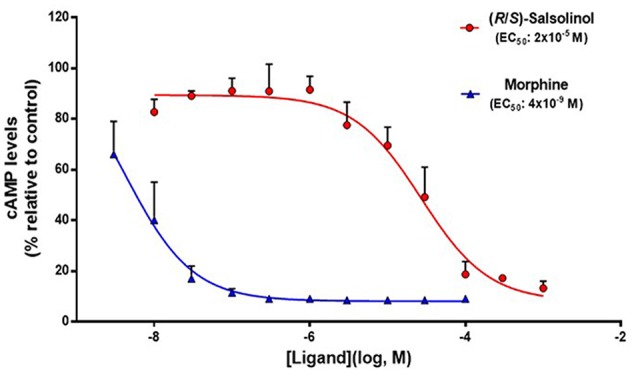
**Salsolinol (SAL) acts as an agonist of μ-opioid receptors.** Functional assay of μ-opioid receptor dose-response activation by morphine and racemic (R/S)-SAL. Each ligand was assayed three times in duplicate using concentrations ranged from 1 × 10^−8^ M to 1 × 10^−3^ for (R/S)-SAL, and 3 × 10^−9^ M to 1 × 10^−4^ M for morphine. Values are expressed as the means of cyclic adenosine monophosphate (cAMP) levels (percentage relative de control) ± SEM. The luminescence is proportional to the intracellular cAMP levels (induced by forskolin); therefore, a decrease in cAMP levels signals the activation of the μ-opioid receptor, which is coupled to a inhibitory G protein (Gi). Half-maximal effective concentration (EC_50_) corresponds to the concentration of ligand eliciting 50% of the maximal response. (R/S)-SAL curve: degrees of freedom, 26 (four points excluded as outliers, at [Ligand](log, M) = −3, −3.5, −7, −7.5); *R*^2^ = 0.8809. Morphine curve: degrees of freedom, 17; *R*^2^ = 0.8825.

To study the capacity of SAL to activate the μ-opioid receptor, we used a commercial assay based in CHO-K1 cells expressing the human μ-opioid receptor. Since the μ-opioid receptor is coupled to an inhibitory G protein (Gi), its activation by an agonist results in a reduction of intracellular levels of cAMP. Figure 1 shows the relative intracellular cAMP levels, measured as a chemiluminescent signal, elicited by the action of different concentrations of racemic SAL and the μ-opioid agonist morphine. Results showed that SAL is effective to activate the μ-opioid receptor through the G protein-signaling pathway but at lower potency compared to morphine. An EC_50_ for racemic SAL was 2 × 10^−5^ M (*R*^2^ = 0.86), while the EC_50_ for morphine was 4 × 10^−9^ M (*R*^2^ = 0.96).

**Figure 2:**
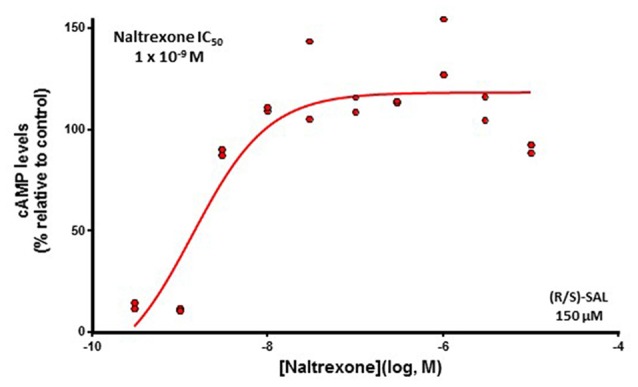
**The action of racemic SAL is fully blocked by the μ-opioid receptor antagonist naltrexone.** Levels of cAMP (percentage relative to control) detected after incubation with racemic (R/S)-SAL 150 μM in the presence of different concentrations of the antagonist naltrexone (1 × 10^−5^ M to 3 × 10^−10^ M) are shown. The activation of the μ-opioid receptor by the addition of the (R/S)-SAL 150 μM was assayed 30 min after the addition of the antagonist. This concentration of (R/S)-SAL elicits 80% of the maximal response of the system. The antagonism of the inhibitory action of SAL on μ-opioid receptor results in an increase of intracellular cAMP levels. The shown results are from one experiment performed in duplicate and each point represents one of the two replicates for each concentration of antagonist. Half-maximal inhibitory concentration (IC_50_) corresponds to the concentration of antagonist reducing 50% the maximal response to the agonist. Degrees of freedom, 7; *R*^2^ = 0.8550.

Following the pharmacodynamic characterization of SAL on the μ-opioid receptor, the effect of different concentrations (1 × 10^−5^ M to 3 × 10^−10^ M) of the antagonist naltrexone was determined on the activation of the μ-opioid receptor elicited by racemic SAL at a concentration of 1.5 × 10^−4^ M. As it is seen in Figure 1, this SAL concentration is able to elicit 80% of the maximal response in this cell-based assay. Figure 2 shows that naltrexone antagonizes, in a dose-dependent fashion, the ability of racemic SAL to activate the μ-opioid receptor and to reduce intracellular cAMP levels. A complete antagonism of the SAL action is reached at naltrexone concentrations of 10^−8^ M. A non-linear fit analysis of the data revealed that naltrexone showed an IC_50_ of 1 × 10^−9^ M (*R*^2^ = 0.85).

### Separation and Purification of (R) and (S)-Salsolinol

**Figure 3:**
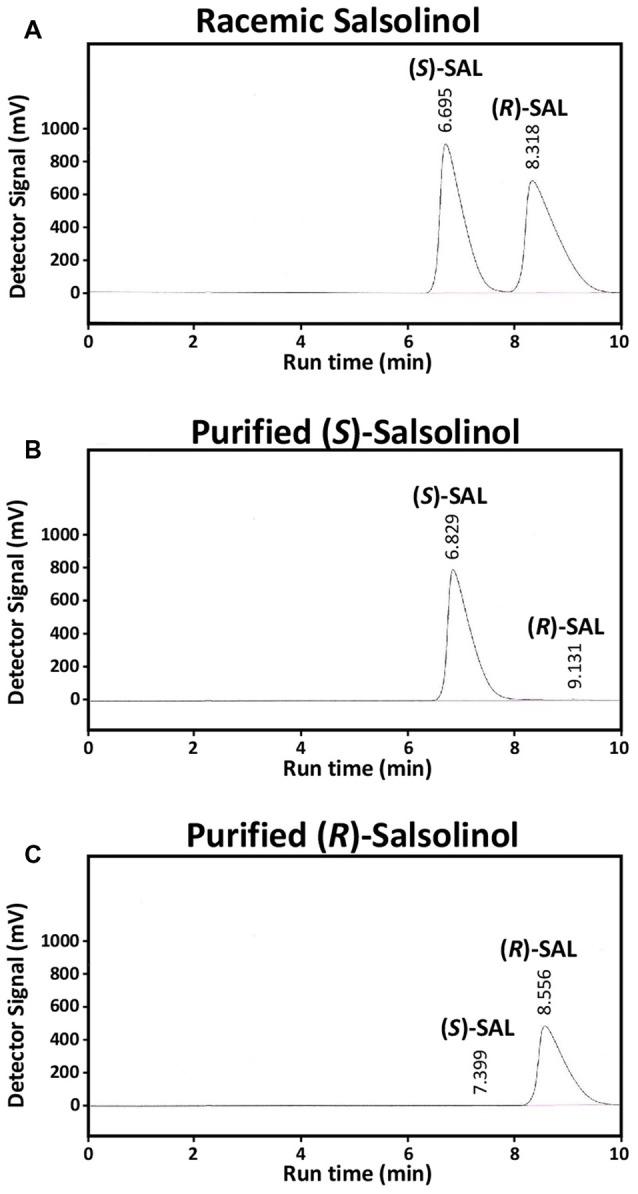
**Purification of (R)-SAL and (S)-SAL from racemic (R/S)-SAL.** High-pressure liquid chromatography (HPLC) chromatographs of **(A)** racemic (R/S)-SAL 1 × 10^−2^ M (corresponding to 5 × 10^−3^ M of each enantiomer), **(B)** purified (S)-SAL 4 × 10^−3^ M (non-detectable levels of (R)-SAL) and **(C)** purified (R)-SAL 3 × 10^−3^ M (non-detectable levels of (S)-SAL). The volume of each sample was 50 μL, and the flow rate was 0.8 ml/min. The concentrations of the enantiomers in the purified solutions were determined using racemic (R/S)-SAL as standard.

The enantiomers (R)-SAL and (S)-SAL were separated and purified from racemic (R/S)-SAL by HPLC. Figure 3A shows the retention time of the enantiomers: (S)-SAL = 6.7 min and (R)-SAL = 8.3 min, in chromatographs showing equal areas for (S)-SAL and (R)-SAL in a sample containing racemic (R/S)-SAL. Figures 3B,C show the chromatograms obtained for purified (S)-SAL and (R)-SAL respectively. The quantification of the area under the curve for (R)-SAL and (S)-SAL showed that both stereoisomers were 99% pure.

### Activation of μ-Opioid Receptors by (R)- and (S)-SAL through the Gi Protein-Signaling Pathway

**Figure 4:**
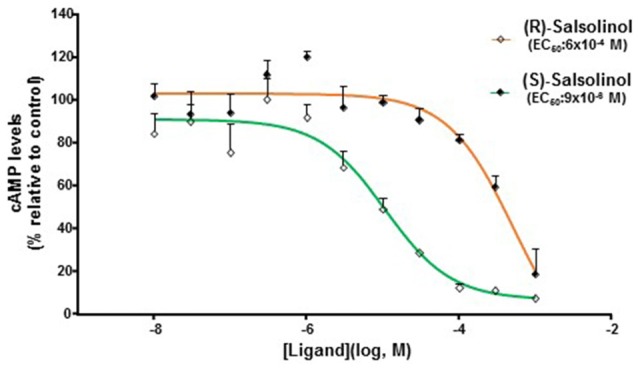
**(R)-SAL and (S)-SAL stereoisomers act as agonists on μ-opioid receptor.** Functional assay of the μ-opioid receptor dose-response activation by (R)-SAL and (S)-SAL. Each ligand was assayed three times, each concentration, in duplicate using concentrations ranging from 1 × 10^−8^ M to 1 × 10^−3^ M. Values are expressed as mean cAMP levels (percentage relative de control) ± SEM. The luminescence is proportional to the intracellular cAMP levels (induced by forskolin); therefore, a decrease in cAMP levels signals the activation of the μ-opioid receptor, which is coupled to a Gi protein. EC_50_ corresponds to the concentration of ligand eliciting 50% of the maximal response. (R)-SAL curve: degrees of freedom, 28 (two points excluded as outliers, at [Ligand](log, M) = −7, −7.5); *R*^2^ = 0.8267. (S)-SAL curve: degrees of freedom, 28 (two points excluded as outliers, at [Ligand](log, M) = −7, −7.5); *R*^2^ = 0.9072.

The capacity of the purified enantiomers (R)-SAL and (S)-SAL to activate the μ-opioid receptor (G protein signaling pathway) was assayed using the same methodology used for assaying racemic SAL. Figure 4 shows the changes in cAMP levels, measured as a chemiluminescent signal, elicited by different concentrations (10^−3^ M to 10^−8^ M) of (R)-SAL and (S)-SAL. Results showed that both enantiomers were effective in activating the μ-opioid receptor, displaying a similar efficacy (capacity to reduce intracellular cAMP levels) but a higher potency (lower concentration needed to activate the receptor) for (S)-SAL compared to (R)-SAL. The EC_50_ for (S)-SAL (9 × 10^−6^ M, *R*^2^ = 0.81) was 50 times lower than the EC_50_ calculated for (R)-SAL (6 × 10^−4^ M, *R*^2^ = 0.71).

### Activation of μ-Opioid Receptors by Racemic SAL through the β-Arrestin Signaling Pathway

Several opioid agonists can activate with different efficacies the G protein-independent signaling pathway led by the recruitment of β-arrestin. This action of opioid agonists has been correlated with their capacity to induce internalization/recycling of μ-opioid receptors (see Williams et al., 2013). For this reason, we studied the effect of different concentrations of racemic SAL and the μ-opioid agonists DADLE and morphine on CHO-K1 cells engineered to express the μ-opioid receptor and to detect the recruitment of β-arrestin in response to such agonists.

**Figure 5:**
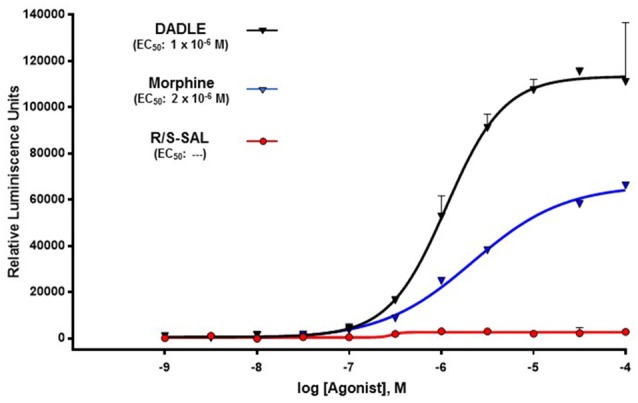
**The action of racemic SAL on the μ-opioid receptor does not induce the recruitment of β-arrestin.** The recruitment of β-arrestin in response to morphine, [DAla^2^, D-Leu^5^]-Enkephalin (DADLE), and racemic SAL was measured using a PathHunter^®^ eXpress β-Arrestin G protein-coupled receptor (GPCR) Assay (DiscoverX). DADLE and SAL were assayed in duplicate using concentrations ranged within 1 × 10^−9^ to 1 × 10^−4^. Data for morphine correspond to a single assay using concentrations ranged within 1 × 10^−8^ to 1 × 10^−4^ M for morphine. Values are expressed as the mean of relative luminescence units (RLU) ± SEM. The luminescence is directly proportional to the recruitment of β-arrestin. EC_50_ corresponds to the concentration of ligand eliciting 50% of the maximal response. DADLE curve: degrees of freedom, 18; *R*^2^ = 0.9838. Morphine curve: degrees of freedom, 6; *R*^2^ = 0.9956.

Figure 5 shows that racemic SAL was, at all tested concentrations, unable to activate the β-arrestin signaling pathway upon its binding on the μ-opioid receptor. In contrast, DADLE showed the highest efficacy in activating the β-arrestin signaling pathway on μ-opioid receptors, displaying an EC_50_ of 1 × 10^−6^ M (*R*^2^ = 0.98). Results also showed that morphine activates the β-arrestin pathway upon its action on μ-opioid receptors (EC_50_ = 2 × 10^−6^, *R*^2^ = 0.99) but displaying a lower efficacy (50%) compared to DADLE. Since racemic SAL was inactive on the β-arrestin signaling pathway, no studies with (R)-SAL and (S)-SAL were conducted on β-arrestin recruitment determination (see “Discussion” Section).

### Molecular Docking of (R)-SAL and (S)-SAL Enantiomers on the μ-Opioid Receptor

To further support the pharmacodynamics findings, molecular docking analyses of morphine, (R)-SAL and (S)-SAL on the coordinates of the binding site of the crystallized mouse μ-opioid receptor were performed. Molecular docking studies started by searching the spatial orientations (pose) of morphine, (R)-SAL and (S)-SAL allowing: (i) the lowest minimum global energy (score) of the ligand-receptor interaction; and (ii) the shorter interaction distance (<4 Å) between the amino group of the ligand and one of the side-chain oxygens of the Asp147 (N-Asp147) residue of the mouse μ-opioid receptor. This interaction has been reported as an important feature for opioid agonist activity (Li et al., 1999; Shim et al., 2013; Huang et al., 2015). The scores and N-Asp147 distances for nine different poses of morphine, (R)-SAL and (S)-SAL, hierarchized according to their minimum score, are showed in Table 1. Morphine displayed the lowest scores among the three tested ligands, consistent with its higher potency for the activation of the μ-opioid receptor. The docking scores for SAL enantiomers were higher than those of by morphine; however, slightly more favorable parameters were found for (S)-SAL, which also showed better scores in all the poses than (R)-SAL.

**Table 1:** **Docking parameters for morphine, (R)-SAL and (S)-SAL on the binding site of the mouse μ-opioid receptor**.

Pose	Morphine		(*R*)-SAL		(*S*)-SAL	
	Score (kcal/mol)	N-O:Asp (Å)	Score (kcal/mol)	N-O:Asp (Å)	Score (kcal/mol)	N-O:Asp (Å)
**1**	−9.7	2.9	−7.5	3.0	−7.7	3.1
**2**	−8.7	6.1	−6.6	3.7	−6.7	5.5
**3**	−8.6	5.0	−6.5	6.2	−6.6	3.3
**4**	−8.4	7.2	−6.5	6.6	−6.5	5.7
**5**	−8.4	2.8	−6.5	6.3	−6.5	3.2
**6**	−8.1	5.5	−6.4	3.2	−6.4	8.4
**7**	−7.4	5.4	−6.4	6.0	−6.3	5.8
**8**	−6.8	7.2	−6.4	7.8	−6.3	7.6
**9**	−6.8	3.4	−6.2	8.0	−6.2	6.6

*Docking scores and Ligand:N-O:Asp147 distances for the poses obtained for morphine, (R)-SAL and (S)-SAL on the binding site of the mouse μ-opioid receptor. The table shows the scores and N-Asp147 distances for nine different poses of morphine, (R)-SAL, (S)-SAL hierarchized according to its minimum score. Poses and its corresponding score were determined using the Autodock Vina software. Scores are expressed as kcal/mol. The predicted distances between the amino group of the ligand and the α-carboxylic acid group of Asp147 (Ligand:N-O:Asp147) were determined using the PyMOL software. Ligand:N-O:Asp147 distances are expressed in Angstroms*.

**Figure 6:**
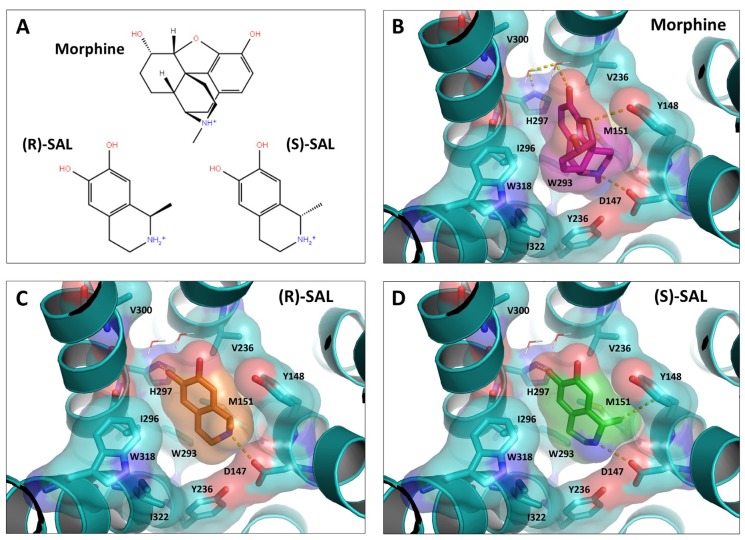
**(R)-SAL and (S)-SAL stereoisomers showed a morphine-like interaction with the binding site of the μ-opioid receptor.** Molecular docking analyses of morphine, (R)-SAL and (S)-SAL on the coordinates of the crystallized mouse μ-opioid receptor were performed. **(A)** Chemical structure of the molecules studied: morphine, (*R*)-SAL and (*S*)-SAL. Hydroxyl groups are represented in red and amino groups are represented in blue. **(B–D)** Best docking fits of the three studied molecules on the binding site of the μ-opioid receptor. The molecular surface of each molecule and the receptor is represented. A dotted line highlights the main interactions of the ligands with the binding site of the μ-opioid receptor.

According to crystallographic studies reported by Shim et al. (2013) and Huang et al. (2015), the residues of the binding site of the μ-opioid receptor that are important for the binding of agonists are Asp147, Tyr148, Met151, Val236, Trp293, Ile296, His297, Val300, Trp318, Ile322 and Tyr326. Figure 6 shows a molecular representation that highlights the interactions of morphine, (R)-SAL and (S)-SAL, arranged accordingly to their best pose, with the aforementioned amino acidic residues. Figure 6B shows that morphine establishes a salt bridge with Asp147 residue, forms hydrogen bonds with Tyr148 and, through two water molecules, with His297. The hydrophobic surface of morphine also binds to the hydrophobic domain of the binding site, formed by Val300, Ile296 and Ile322. Figures 6C,D show the molecular interactions predicted for (R)-SAL and (S)-SAL, respectively. Since SAL enantiomers are smaller molecules compared to morphine, they establish fewer interactions with the receptor. Figure 6C shows that (R)-SAL forms a salt bridge with Asp147 and, unlike morphine, the orientation of its hydroxyl groups allows a direct hydrophobic bond with His297. The hydrophobic surface of (R)-SAL also interacts with Ile296 and Met151. Figure 6D shows that the binding of (S)-SAL with the μ-opioid receptor is very similar to the one showed by (R)-SAL, predicting interactions with Asp147 and His297, and hydrophobic interactions with Ile296 and Met151. However, the spatial orientation of the methyl group, that defines the difference between the two SAL enantiomers, contributes to an additional interaction of (S)-SAL with Tyr148, which is not present in the simulation for the (R)-SAL enantiomer.

## Discussion

The findings of this study support the hypothesis that SAL acts as an agonist on the μ-opioid receptor. Overall, we found that SAL is effective in activating the μ-opioid receptor, showing an EC_50_ of 2 × 10^−5^ M. The agonist activity of SAL on the μ-opioid receptor was fully blocked by the μ-opioid antagonist naltrexone. Purified (R)-SAL and (S)-SAL stereoisomers showed to be effective in activating the μ-opioid receptor, displaying an EC_50_ of 6 × 10^−4^ M and 9 × 10^−6^ M respectively. In agreement with these results, molecular docking simulations predicted a morphine-like interaction of (R)-SAL and (S)-SAL stereoisomers with the μ-opioid receptor and a favored interaction for the (S)-SAL stereoisomer.

The assays aimed at determining the intrinsic activity of SAL showed that SAL is, as the full agonist morphine, effective in activating the μ-opioid receptor, since both compounds display nearly the same ability to inhibit the generation of intracellular cAMP (as shown by a reduction in chemiluminiscence in the present cell study). However, a marked difference was observed between morphine and SAL on their potency, since the EC_50_ of SAL was 5000-fold higher than that of morphine. These results are in agreement with early radioligand binding studies performed in rat striatum that showed that SAL can displace the μ-opioid receptor agonist [met]-enkephalin with an IC_50_ of 10 μM (1 × 10^−5^ M; Lucchi et al., 1982). Recent electrophysiological studies in slices of posterior VTA have shown that incubation with SAL (10^−8^ M to 10^−6^ M) results in an increased firing of dopamine neurons. These stimulatory effects of SAL on dopaminergic neurons are abolished by the addition of naltrexone (Xie et al., 2012). As proposed by Xie et al. (2013), SAL can indirectly activate dopaminergic neurons in the VTA through the inhibition of local GABAergic interneurons controlling the activity of dopaminergic neurons, playing a key role on the rewarding responses to SAL exposure. Recent *in vivo* studies have determined the effects of SAL on the activity of dopaminergic neurons in the VTA. Microdialysis studies in Wistar rats have shown that the intra-VTA administration of SAL at concentrations ranging within 10^−7^ M to 10^−4^ M can elicit a marked increase of dopamine release in the nucleus accumbens (Hipólito et al., 2011; Deehan et al., 2013). In addition, the microinjection of SAL into the VTA of Wistar rats at concentrations of 150 μM (15 × 10^−5^M) resulted in a significant induction of locomotor activity (Hipólito et al., 2010), conditioned place preference (Hipólito et al., 2011) and ethanol intake (Quintanilla et al., 2014). These results show that the concentrations at which SAL exerts its neurochemical and behavioral effects in the VTA are similar to those able to activate μ-opioid receptors in the present *in vitro* study (EC_50_ = 2 × 10^−5^ M).

In support of an agonistic action of SAL on μ-opioid receptor, we found that the opioid antagonist naltrexone was able to block almost completely the action of SAL with an IC_50_ of 1 × 10^−9^ M. These values of IC_50_ for naltrexone are in agreement with previous reports regarding the potency of naltrexone as an antagonist of the μ-opioid receptor (Hahn et al., 1985; Michel et al., 1985). These findings are also in line with evidence showing that SAL effects are blocked by μ-opioid receptor antagonists; e.g., conditioned place preference (Matsuzawa et al., 2000); *in vitro* dopaminergic neurons activation (Xie et al., 2012); dopamine release in the nucleus accumbens (Hipólito et al., 2011); locomotor activation (Hipólito et al., 2010) and increased ethanol consumption (Quintanilla et al., 2014). An alternative mechanism by which SAL could activate opioid transmission is by increasing the release of endogenous opioids, such as β-endorphins. However, *in vitro* studies on primary cultures of hypothalamic neurons have shown that SAL, contrary to ethanol and acetaldehyde, is unable to stimulate the secretion of β-endorphins (Reddy and Sarkar, 1993).

The classic signaling pathway associated to the agonist-mediated activation of GPCRs involves the activation of the corresponding heterotrimeric G protein and phosphorylation of intracellular domains of the GPCR by G protein-coupled receptor kinases (GRKs), which results in rapid desensitization of GPCR to agonist action. Upon phosphorylation of GPCRs, a conformational change greatly increases its affinity to β-arrestin, a protein involved in receptor endocytosis processes and activation of signaling pathways leading to different cellular responses. After arrestin-mediated endocytosis, GPCR can be either degraded or dephosphorylated and recycled to the membrane surface as functional receptors (resensitization; See Williams et al., 2013). For some GPCRs, including μ-opioid receptors, there are agonists displaying a differential efficacy (biased agonists) for recruiting β-arrestin proteins (Kenakin, 2011). In this study, we found that SAL acts as a biased agonist on μ-opioid receptors, since it activates efficiently the signaling pathway led by Gi protein, but not the recruitment of β-arrestin. Interestingly, it has been proposed that agonists that do not efficiently recruit β-arrestin, and therefore are not able to promote an effective receptor endocytosis, may have an increased potential to produce tolerance and dependence due to a reduction of functional receptors (Whistler et al., 1999; Finn and Whistler, 2001).

Taking into account the relatively low potency showed by (R/S)-SAL in activating the μ-opioid receptor (EC_50_ = 2 × 10^−5^ M), the question that arises is if the concentration of SAL generated in the brain after ethanol consumption is enough to generate an opioid response. Despite several animal studies showing that chronic administration of ethanol increases SAL levels in the hypothalamus and the striatum (Sjöquist et al., 1982; Myers et al., 1985; Matsubara et al., 1987), there are no studies showing the actual concentrations of SAL generated in the brain by acute pharmacological doses of ethanol. Brain microdialysis studies by Jamal et al. (2003) showed that SAL is only detectable at nanomolar levels in the striatum of rats whether ethanol administration is preceded by a pre-treatment with the aldehyde dehydrogenase inhibitor cyanamide, thus increasing acetaldehyde levels. These results indicate that *in vivo* formation of SAL is highly dependent on acetaldehyde levels, occurring only in dopamine-rich areas of the brain. Since catalase is the main enzyme responsible for the metabolism of ethanol to acetaldehyde in the brain, a heterogeneous distribution of catalase may allow the generation of site-specific accumulations of acetaldehyde, yielding high local concentrations of SAL (see Hipólito et al., 2007). In agreement to this, studies by Brannan et al. (1981) showed that catalase activity displays a regional distribution in the rat brain, with high activity in the midbrain, a dopamine-rich area that contains the VTA, deeply involved in the rewarding effects of ethanol.

An alternative ethanol-independent route of SAL biosynthesis in the brain has been proposed, involving the condensation of dopamine with pyruvic acid to yield an intermediate metabolite (salsolinol-1-carboxylic acid), which can be converted in SAL through an enzymatic oxidative decarboxylation (Naoi et al., 1996). However, experimental evidence regarding the identity and properties of the enzymes involved in this suggested biosynthetic pathway are still lacking.

The analysis of the actions of the (R) and (S) stereoisomers of SAL showed that both molecules act as full agonists of the μ-opioid receptor, with (S)-SAL being 50-fold more potent than (R)-SAL. In line with a putative role of (S)-SAL on the actions of ethanol, human studies by Rommelspacher et al. (1995) showed that (S)-SAL levels in the plasma of alcoholics are 100-fold higher than that in non-alcoholics, while (R)-SAL levels in the plasma of alcoholics are only 2-fold higher than that in non-alcoholic subjects. An animal study performed in alcohol-preferring P rats showed that after 8 weeks of chronic ethanol consumption, there was a 2-fold increase in (S)-SAL levels in the midbrain, whereas a 1.6-fold increase was detected in (R)-SAL levels (Rojkovicova et al., 2008). In contrast to these findings, a recent behavioral study by Quintanilla et al. (2016) found that (R)-SAL was the only enantiomer capable of inducing conditioned place preference after its intracerebral infusion into the VTA of rats. The reasons for these inconsistencies are unknown, but a possible explanation would be the existence of different molecular targets for (R)-SAL or its *in vivo* metabolism. Furthermore, there are studies showing that SAL increases monoaminergic transmission by inhibition of monoamine oxidase (MAO) activity in the brain (Naoi et al., 2004) and by inhibition of monoamine reuptake (Alpers et al., 1975). Recent studies have also shown that SAL could interact with other receptors as a monoamine derivative. Electrophysiological studies by Xie and Ye (2012) showed that the stimulant effect of SAL on dopaminergic neurons could be attenuated by the D1 receptor antagonist, SKF83566. In fact, intracerebral self-administration of SAL in alcohol-preferring P rats is significantly reduced by the co-administration of the dopamine D_2,3_ agonist quinpirole or the serotonin 5-HT_3_ antagonist ICS-205, 930 (Rodd et al., 2008). It is also possible that both (R)-and (S)-SAL could present different affinities for other opioid receptors such as the kappa and delta subtypes.

The higher potency showed by (S)-SAL is also supported by molecular docking studies in which the binding of each stereoisomer to the μ-opioid receptor was simulated and analyzed. As shown in Table 1, in spite of the similarity of the two enantiomers, (S)-SAL docking poses had lower scores (predicted binding energy) than (R)-SAL in general, and also when comparing best poses, which could account for its higher experimental agonist potency. The low score of (S)-SAL can be explained by the interaction of its chiral methyl group with the receptor binding site, specifically with the Tyr148 (Figure 6D), filling a cavity that is empty for (R)-SAL (Figure 6C). To our knowledge SAL (179 Da) is the smallest molecule described to date showing an opioid full agonism. A possible limitation for the present docking study is that computational simulations were performed using the crystal structure of the mouse μ-opioid receptor instead of the human μ-opioid receptor used in the cell-based assays. However, an overall comparison of the amino acid sequence shows a 94% of homology between the mouse and human receptors. A near complete homology (>99%) is obtained if the comparison only considers the domains comprising the binding site of the receptor.

Overall, it is shown that racemic (R/S)-SAL and its stereoisomers (R)-SAL and (S)-SAL are agonists of the μ-opioid receptor, (S)-SAL being more potent than (R)-SAL. The *in silico* study also shows that the interactions of (R)-SAL and (S)-SAL with the binding-site of the μ-opioid receptor are analogous to that shown by morphine. Further studies of the action of SAL on μ-opioid receptor variants (e.g., A118G variant) or other opioid receptor subtypes (e.g., delta or kappa) would provide additional evidence of the role of SAL in the development of alcohol addiction.

## Author Contributions

PBC, MR-M, and GZ-T conceived and designed the experiments, analyzed the data. PB-C performed the experiments. MR-M, PB-C, MH-M, MEQ, and VV wrote or contributed to the writing of the manuscript.

## Funding

This work was supported by Fondo Nacional de Desarrollo Científico y Tecnológico (FONDECYT) grants #11130241 (MR-M), and #1120079 (MH-M); Comisión Nacional de Investigación Científica y Tecnológica (CONICYT) PCHA/Doctorado Nacional/2013-21130865 (PB-C); National Institutes of Health (NIH) grants AA022057 (VV), and AA021724 (VV); Millennium Institute BNI P09-015-F (MH-M; MR-M).

## Conflict of Interest Statement

The authors declare that the research was conducted in the absence of any commercial or financial relationships that could be construed as a potential conflict of interest.
